# Characterizing the Decision-Making Competency of Nurse Managers: A Scoping Review

**DOI:** 10.1155/jonm/2771210

**Published:** 2025-06-10

**Authors:** Alberto Gonzalez-Garcia, Pilar Marques-Sanchez, Arrate Pinto-Carral, Raquel Leiros-Rodriguez, Javier Perez-Rivera, Silvia Perez-González

**Affiliations:** ^1^Faculty of Health Sciences, Nursing and Physiotherapy Department, HrQoL Research Group, University of Leon, Castile and León, León 24007, Spain; ^2^Faculty of Health Sciences, Nursing and Physiotherapy Department, SALBIS Research Group, University of Leon, Ponferrada Campus, Castile and León, Ponferrada 24002, Spain; ^3^Faculty of Health Sciences, Nursing and Physiotherapy Department, SALBIS Research Group, University of Leon, Castile and León, León 24007, Spain

**Keywords:** competency, decision-making, nurse executive, nurse manager

## Abstract

**Objective:** The purpose of this scoping review is to describe the characteristics of the decision-making competency of nurse managers. In addition, this review analyzes how technological, organizational, and contextual factors influence the development and practical application of this competency and identifies the key barriers and facilitators that specifically impact its growth and use in managerial practice.

**Background:** Nurse managers' decision-making abilities are essential for organizational performance in rapidly evolving healthcare systems. Despite its relevance, the specific characteristics that define this competency have not been thoroughly examined. Following our research methodology, we searched the Web of Science, Scopus, CINAHL, and PubMed electronic databases, focusing on the decision-making characteristics of nurse managers between January 2009 and February 2025.

**Results:** Twenty-five studies were included, identifying 15 core characteristics that define nurse managers' decision-making competency. The most frequently reported characteristics were professional values and ethics, information processing, systems and data management, and risk management. Technology was consistently identified as a critical facilitator of decision-making, enabling efficient information access and analysis. Conversely, high levels of stress and the complexity of healthcare environments were identified as the primary barriers to effective decision-making.

**Conclusions:** Strengthening decision-making competency in nurse managers requires the development of ethical reasoning, data literacy, systems thinking, and risk evaluation. Technological proficiency is essential to enhance decision quality, while organizational stressors must be managed to reduce cognitive load and improve performance.

**Implications for Nursing Management:** These findings support the design of targeted training programs, particularly those using simulation and virtual reality, focused on strengthening decision-making competency, a critical skill for effective nursing management. Developing this competency enhances nurse managers' ability to navigate complex clinical and organizational challenges, make ethically sound decisions, and lead their teams with greater confidence and strategic insight.

## 1. Introduction

Due to the continuous changes in healthcare systems, efficient decision-making is recognized as necessary to achieve the objectives set at different levels of management and ensure their sustainability [1–3]. Decision-making is a crucial competency for managers in any institution, including healthcare settings [4–6], to address institutional problems, both in the long and short term, with novel, creative, and efficient solutions [7].

In healthcare management, decision-making is a multifaceted process that occurs at various levels, individual, team, and organizational, and can have both temporary and long-lasting effects [8, 9]. Most problems comprise multiple interrelated factors, so decision-making must be approached as a complex and dynamic task [10]. More importantly, all aspects must be considered to arrive at a sound diagnosis of the problem and select the best alternative among the possibilities that arise [11–13]. Therefore, deciding involves taking a course of action that includes understanding the problem, identifying factors, developing alternatives, selecting the best alternative, implementing the sequence of activities to be performed, and establishing a control and evaluation system [4, 14].

Nurse managers are responsible for planning and organizing nursing services, ensuring safe and healthy care environments, supporting teamwork, engaging patients, and evaluating service outcomes to promote efficiency [15, 16]. Their leadership role is fundamental to organizational success, influencing patient care quality, staff satisfaction, economic sustainability, and strategic goal achievement [17, 18].

There are three functional roles in nurse management: (1) Operational-level nurse managers, who oversee daily clinical operations and direct patient care activities within specific clinical units; (2) logistical-level nurse managers, who manage resources, coordinate interunit activities, and supervise departmental or area-level functions; and (3) executive-level nurse managers, who hold strategic responsibilities at an organizational level, guiding policy development and organization-wide strategy implementation [19]. At these three levels, nurse managers play a significant role in the success of the organization, given that they establish the strategies that guide nurses in care practice; supervise day-to-day activities; support the organization's development by leading different processes that encompass planning, resource, and people management; and formulate strategies that influence health policies in a changing healthcare environment [18, 20, 21]. Therefore, nurse managers must develop core competencies to effectively perform these functional roles, including decision-making, relationship management, communication skills, active listening, leadership, conflict management, ethical principles, collaboration, and teamwork [22, 23]. González-García [3] emphasizes that decision-making competency is essential for nurse managers across all functional levels, operational, logistical, and executive. This scoping review adopts a comprehensive perspective, examining decision-making competency as a core requirement at all levels of nurse management.

Consequently, the decisions made by nurse managers not only impact the structure and functioning of the institution but also influence the activity of professionals and, consequently, patient care [24, 25]. However, decision-making is not always straightforward, especially in a volatile healthcare environment characterized by rapid technological change and evolving patient needs [26]. Managing such an environment is a nuanced and challenging task, requiring tacit knowledge that is not only predominantly acquired through contextual experience but can also be supported through training and simulation environments [27]; therefore, the ability to make decisions emerges as a key competency for nurse managers [3].

Although decision-making has been widely discussed in the management literature, the decision-making process in nursing management remains challenging to determine and understand [8, 28]. Existing studies have typically focused on decision-making styles, processes, or outcomes in clinical and managerial contexts, emphasizing either rational–analytical approaches or intuitive strategies developed through experience. However, these studies often lack a clear conceptualization of decision-making as a competency, an integrated set of knowledge, skills, and attitudes that enable nurse managers to make effective decisions. In particular, no research has systematically identified or defined the specific characteristics that constitute decision-making competency in nurse managers. Since these characteristics represent the core elements through which this competency is developed and demonstrated in practice, this review aimed to identify and describe them in detail. To further deepen our understanding of this competency, we also analyzed how technological, organizational, and contextual factors specifically characterize decision-making competency for nurse managers by shaping their abilities, influencing their decision-making processes, and defining key attributes. In addition, we sought to identify the main barriers and facilitators that directly impact this competency's practical expression and development.

In the context of nurse management, “competency” refers to an integrated set of knowledge, skills, attitudes, and behaviors required to perform management tasks [23] effectively. In this study, “decision-making” is the process through which nurse managers assess problems, interpret relevant data, weigh ethical considerations, evaluate alternatives, and take timely actions to guide both clinical teams and nursing outcomes [29]. This process combines analytical reasoning, clinical judgment, and moral responsibility within dynamic and high-pressure healthcare environments [30]. Accordingly, decision-making competency involves the nurse manager's ability to analyze complex situations, synthesize information, and make informed, ethical, and strategic decisions [31].

The characteristics described in this review represent the observable elements of decision-making competency in practice [32]. These include analytical thinking, ethical judgment, effective communication, and resilience under pressure, each contributing uniquely to decision-making proficiency [33]. While competencies encompass broader, integrative capabilities that evolve with experience and context, characteristics are the behavioral indicators that inform both development strategies and performance assessment in nursing management [23].

## 2. Materials and Methods

### 2.1. Design

A scoping review integrates quantitative and qualitative research findings to address a specific question. This approach is beneficial for identifying new evidence, recognizing knowledge gaps, and applying structured, transparent methods to map and analyze existing literature [34]. In addition, scoping reviews facilitate systematic and meaningful data extraction [35]. This review adheres to the PRISMA-ScR checklist and follows the methodological framework established by Moher et al. [36]. It was conducted according to the approach proposed by Arksey and O'Malley [34], which involves the following steps: identifying the research question, identifying relevant studies, selecting those studies, extracting relevant data, and summarizing and reporting the findings.

Furthermore, this study replicated the methodology established by González-García et al. [23], incorporating a search strategy, article selection criteria, and data extraction and coding procedures. This methodology was chosen because it aligns with the ongoing line of research aimed at characterizing and operationalizing the competency model for nurse managers, initially developed by González-García [19]. Ensuring methodological consistency is crucial to maintaining the coherence of research findings. In addition, this process is registered in the Intellectual Property Registry (no. 00/2024/3056) of the Ministry of Culture in Spain.

### 2.2. Research Question Identification

The following research questions were formulated to guide the scoping review:

1. What are the characteristics of nurse managers' decision-making competency?
2. How do technological, organizational, and contextual factors shape and influence the development and application of decision-making competency among nurse managers?
3. What are the main barriers and facilitators that influence this competency?

### 2.3. Search Method

Articles published between 2009 and 2025 were searched across electronic databases, including Web of Science, Scopus, and PubMed, using relevant keywords related to nurse managers and decision-making competency (Table 1).

During the planning phase of this review, we considered a wide range of search terms, including technology, barriers, and facilitators, among others. However, a preliminary analysis of the retrieved articles revealed that many studies under these broader terms addressed decision-making in a general sense but did not focus on decision-making competency or its defining characteristics. Since our primary objective is to characterize decision-making competency rather than to examine broader decision-making processes, we refined the search strategy to improve relevance. Consequently, we excluded specific terms that, while conceptually related, yielded results that did not align with the particular focus on competency characterization required for this study. This decision ensures that the included studies contribute directly to identifying the key characteristics that define decision-making competency in nurse managers.

### 2.4. Study Selection

The inclusion criteria for the articles in this scoping review included quantitative, qualitative, and mixed-methods studies, theses, and dissertations. Articles published in English or Spanish between February 2009 and January 2025 were included to ensure the incorporation of current knowledge. This time frame was chosen to encompass the development of knowledge regarding the role of nurse managers and decision-making within this period.

Conference abstracts, editorials, and discussion papers were excluded, as were articles that did not include data on nurse managers' decision-making characteristics.

**Figure 1:**
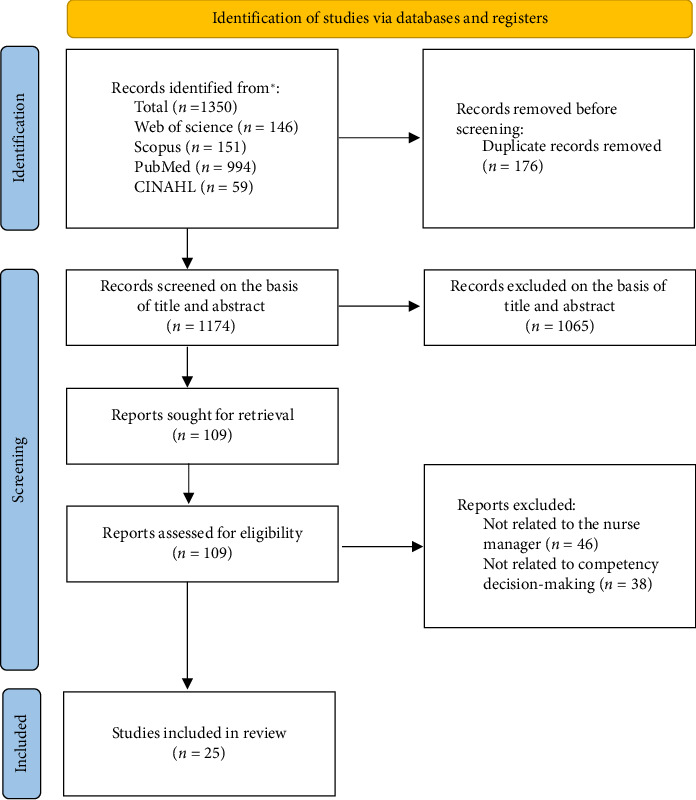
PRISMA flowchart.

The selection process followed PRISMA-ScR guidelines, with independent screening and consensus resolution. The Results section provides a detailed summary of search outcomes, illustrated in Figure 1.

### 2.5. Quality Assessment of Articles

It is essential to clarify that the quality assessment did not serve as an exclusion criterion but was intended to evaluate the overall rigor of the included studies, ensuring a transparent analysis of the existing knowledge base.

Since no fully validated tools were available to assess the diverse methodologies of the included studies, we adapted an existing framework. Specifically, we modified parts of the method proposed by Hölbl et al. [37] to tailor the assessment criteria to the scope of this study. The quality assessment process involved several steps. First, one author initially evaluated each study using the adapted criteria. Then, five additional reviewers independently assessed the same studies to ensure reliability. Each study was scored on a scale from 0 to 2 for each quality criterion, and any discrepancies were discussed and resolved through consensus. The final scores were averaged across reviewers to provide a comprehensive quality evaluation (see Table 2).

### 2.6. Data Extraction

Data extraction was conducted using structured forms to ensure accuracy and consistency. The following information was systematically extracted from each study: study type, sample size, participant characteristics, country of study, and decision-making characteristics. Data analysis was performed using Microsoft Excel.

To systematically code the extracted information, the following procedure was applied:

- Articles were coded with the letter “A” followed by three digits, starting with A001 for the first article.
- Decision-making characteristics were coded with the letter “C” followed by three digits, starting with C001 for the first characteristic.

For instance, the code A003 C007 corresponds to characteristic seven extracted from Article 3.

Using this approach, we initially cataloged the decision-making characteristics identified in the articles and assigned each a unique identifier. These characteristics were then analyzed and grouped based on conceptual similarities, and their frequency of occurrence was recorded. Any discrepancies were discussed and resolved among the reviewers.

In this study, “decision-making characteristic” refers to the elements that reflect how nurse managers engage in decision-making. These characteristics were grouped into emergent themes to facilitate thematic analysis, enabling a more precise identification of patterns within the broader decision-making competency.

## 3. Results

This review included 25 studies (Table 3). The studies were conducted in the United States (*n* = 5), Brazil (*n* = 3), Saudi Arabia (*n* = 3), Iran (*n* = 2), Australia (*n* = 2), Ethiopia (*n* = 2), and one each in Finland, Indonesia, Bangladesh, China, Philippines Israel, Lithuania, and Türkiye. Nine studies employed a qualitative design [29, 38–45], 13 were quantitative [1, 31, 46–56], and three used mixed methods [8, 57, 58].

The search results are illustrated in Figure 1. A total of 1350 records were identified through four databases: Web of Science (*n* = 146), Scopus (*n* = 151), PubMed (*n* = 994), and CINAHL (*n* = 59). After removing 176 duplicates, 1174 records were screened by title and abstract. Of these, 1065 were excluded. The remaining 109 full-text articles were retrieved for eligibility assessment. After a detailed review, 84 articles were excluded: 46 did not focus on the nurse manager role, and 38 did not address decision-making competency. Ultimately, 25 studies were included in the final review.

### 3.1. Quality Evaluation of Included Studies

This section presents the results of the quality evaluation of the included studies to provide transparency regarding the rigor and validity of the sources analyzed in this scoping review.

The quality of the included studies ranged from moderate to high. The methodological quality of the included studies ranged from moderate to high. The mean total quality assessment score was 8.2 (out of a maximum of 10), indicating acceptable overall rigor across the sample. The corresponding average scores for each item are as follows: Q1 (Is the nurse manager decision-making characteristic described?) = 2 ± 0.00, Q2 (Are the research objectives clearly outlined?) = 1.92 ± 0.39, Q3 (Are the main contributions to nurse management decision-making well described?) = 1.48 ± 0.75, Q4 (How appropriate is the problem-solution fit?) = 1.4 ± 0.63, and Q5 (Are the proposed solutions feasible?) = 1.4 ± 0.75. Challenges were particularly noted in Q4 and Q5, where some studies did not completely align their theoretical insights with practical contexts or proposed solutions that were not immediately applicable. These variances in scores indicate the range of depth and applicability of the reviewed studies. These findings help contextualize the strength of the evidence base used in this study (see Table S1, Supporting File 1).

### 3.2. Description of the Articles Included in the Review

The following table summarizes the articles included in this review. The key findings have been carefully analyzed and refined to ensure alignment with the study's focus on decision-making competency in nursing management. Each article's conclusions are interpreted within the context of decision-making competency, highlighting its defining characteristics, influencing factors, and relevant implications for nursing management (see Table S2, Supporting File 1).

### 3.3. Thematic Categorization of Decision-Making Competency Characteristics

From the initial 168 characteristics identified across the reviewed studies, we systematically grouped them based on conceptual similarities. Those describing the same or closely related concepts were consolidated into broader thematic categories. Each category label reflects the defining theme of the grouped characteristics. For example, the “professional values and ethics” category includes 22 distinct characteristics, all referring to ethical principles guiding nurse managers' decision-making processes. Table 3 presents each category's absolute (*n*) and relative (%) frequencies, allowing for conceptual clarity and a quantifiable overview of the competency's components.

These thematic categories provide a structured representation of the defining elements of nurse managers' decision-making competency. The analysis offers conceptual depth and practical utility by organizing the 168 identified characteristics into conceptually coherent groups. This categorization not only clarifies the core dimensions of the competency but also enables the prioritization of development strategies based on the relative prominence of each category in the literature.

From the analysis of these characteristics, we observe that the most frequently cited attributes in the literature are professional values and ethics (13.1%), professional development and feedback (12.5%), communication and collaboration (10.12%), and information processing (9.52%). These findings suggest that nurse managers' decision-making competency is shaped primarily by a strong ethical foundation, effective information handling, and structured approaches to managing risks and clinical data.

The 15 thematic categories were organized into four overarching dimensions to structure the understanding of decision-making competency further, each representing a core component of the decision-making process. In this study, a dimension refers to a higher-order conceptual grouping integrating related characteristics and categories. It offers a holistic view of how nurse managers develop and apply their decision-making.

These dimensions are not isolated; they are interconnected components supporting informed, ethical, and strategic decisions in complex healthcare environments. This framework highlights its multifaceted structure by enhancing the competency's theoretical clarity and practical applicability (Table 4).

The classification of characteristics into these four dimensions allows for a more structured and functional interpretation of decision-making competency in nurse managers. Strategic and ethical decision-making ensures that managerial decisions align with professional values, moral principles, and adaptive thinking in high-pressure environments, and information synthesis and application support evidence-based decision-making through effective data management and analytical processing. Organizational and interpersonal decision-making emphasizes the role of collaboration, communication, and institutional support in shaping effective managerial decisions. Finally, professional growth and reflective decision-making capture the ongoing development of expertise, confidence, and ethical awareness in decision-making processes.

These interconnected dimensions offer a comprehensive perspective on how decision-making competency is developed and applied in nurse managers' roles, aligning with the broader objectives of leadership and healthcare administration.

Beyond identifying the core characteristics of decision-making competency, it is also essential to understand how these competencies are applied in practice. One way to analyze this is through decision-making styles, which represent the different approaches that nurse managers use when making decisions.  Table S3 in Supporting Information 1 presents the decision-making styles identified in the reviewed studies, illustrating the variability in decision-making approaches and how these styles influence the decision-making process in nursing management.

The findings indicate that nurse managers employ a variety of decision-making styles, ranging from structured and tactical approaches to more intuitive and spontaneous strategies. Ethical decision-making styles underscore the importance of professional values and moral reasoning in managerial roles. In contrast, participative and dependent styles highlight the role of collaboration and institutional hierarchy in decision-making processes. Studies incorporating various styles suggest that decision-making competency is not static but adaptable, influenced by situational demands, organizational culture, and individual managerial experience.

Table S4 in Supporting Information 1 presents an integrated synthesis of contextual and organizational factors, personal attributes, and their documented consequences for patients, staff, and healthcare organizations to comprehensively understand how decision-making competency is shaped and expressed in real-world settings.

The integrated analysis of contextual, personal, and outcome-related variables reveals a multifactorial framework shaping decision-making competency in nurse managers. Organizational culture, structural support, and leadership development programs emerged as key enablers, while ambiguity in managerial roles, inadequate training, and hierarchical constraints acted as persistent barriers. Personal attributes, such as ethical sensitivity, emotional intelligence, and strategic thinking, were consistently linked to improved leadership behaviors and decision quality. Several studies identified a direct connection between managerial decision-making and positive staff outcomes, including increased autonomy, confidence, and role clarity. Although evidence linking these competencies to patient outcomes was limited, studies highlighted improved patient safety and satisfaction in contexts where nurse managers operated within supportive and ethically aligned environments.

Another critical point is how technological tools and systems, such as hospital information systems and electronic health records, support or interfere with decision-making (see Table S5, Supporting File 1).

The results show that technology enhances information management, improving decision-making in complex scenarios. This advancement helps eliminate barriers associated with decision-making processes.

Table S6 in Supporting Information 1 outlines the main barriers, facilitators, and improvement strategies related to nurse managers' decision-making processes to describe how decision-making competency can be supported or constrained in practice.

The findings reveal diverse contextual, organizational, and individual factors facilitating or hindering nurse managers' decision-making competency. Among the most prominent facilitators are ethical sensitivity, leadership environments with institutional support, and access to structured training programs. In contrast, the main barriers include a lack of institutional backing, limited managerial autonomy, cultural conflicts, and insufficient leadership preparation.

The studies in this review consistently underscore the importance of targeted strategies, such as competency-based training, peer mentorship, and leadership development programs, to overcome these limitations. Integrating moral reasoning, emotional intelligence, and cultural competence is key to strengthening decision-making in complex healthcare environments.

**Figure 2:**
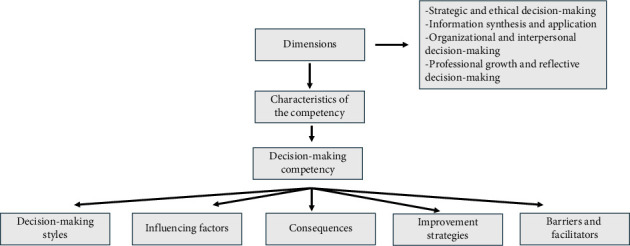
Overview of the scoping review findings. *Source:* own elaboration.

To summarize the key findings of this scoping review, Figure 2 presents a conceptual framework that integrates the central components of nurse managers' decision-making competency. At the core of the model are the specific characteristics that define this competency in practice. These characteristics, identified across the reviewed literature, are grouped into four higher-order dimensions that reflect the main conceptual domains of the competency.

The model surrounds this core with additional analytical components derived from the review, such as decision-making styles, influencing factors, consequences, technological supports, strategies for improvement, and facilitators and barriers. While these elements do not constitute the core of the competency, they provide valuable contextual information that helps explain how it is developed, expressed, and supported in complex healthcare environments.

## 4. Discussion

This review aimed to deepen the understanding of decision-making competency in nurse managers by identifying and interpreting its defining characteristics. Unlike previous studies focusing on decision-making processes or styles, this review consolidates evidence on the specific qualities underpinning decision-making in complex healthcare settings.

This review identified 15 core characteristics, organized into four dimensions, that define decision-making competency in nurse managers. Professional values and ethics, information processing, systems and data management, and risk management emerged as particularly critical. These elements function synergistically, supporting decision-making's technical, ethical, and contextual dimensions in complex healthcare environments.

Specifically, ethical values are foundational to decision-making competency, as they foster accountability, credibility, and trust within teams. Far from being abstract ideals, ethical principles serve as operational guides that shape behavior under pressure and reinforce professional integrity [59]. Nurse managers who consistently demonstrate ethical judgment create psychologically safe environments, enhance team cohesion, and legitimize their leadership, which is essential to effective decision-making in high-stakes scenarios [59–61].

Information processing and data management represent essential characteristics of decision-making competency in nurse managers. These characteristics allow managers to filter and synthesize complex information, reducing cognitive overload and enhancing the precision of decisions made in fast-paced clinical settings [62, 63]. Our findings support the idea that information literacy is not simply a technical characteristic but a strategic one underpinning adaptive, evidence-informed leadership. In line with the strategic information systems theory, data-management characteristics facilitate more efficient decision-making, innovation, and responsiveness in managerial roles [64].

Similarly, integrating risk management within this competency provides nurse managers with tools to navigate uncertainty, assess potential outcomes, and adopt proactive approaches to organizational challenges [65]. Rather than reacting defensively to crises, competent nurse managers leverage risk awareness to anticipate change, mitigate disruption, and lead with foresight [66]. This dimension reinforces that decision-making is not merely cognitive but strategic, anticipatory, and grounded in contextual awareness.

Beyond individual cognitive characteristics, our findings highlight the strategic role of technology in supporting decision-making competency. In contexts where nurse managers face information overload, technological tools, such as decision-support systems and data dashboards, can reduce cognitive burden by organizing and filtering relevant data, thus improving accuracy, speed, and clarity of decisions [67–69]. From this perspective, technology supports information processing and enables collaborative decision-making and proactive risk management within complex organizational structures.

Complementing this technological dimension, the human aspect of decision-making remains equally critical. The variety of decision-making styles identified in the literature reflects the adaptability required in diverse healthcare environments. This aligns with contingency theory, which posits that effective decision-making depends on the fit between style and situational context [70, 71]. In this sense, flexible and context-sensitive decision-making styles are not peripheral but integral expressions of competency. They enhance responsiveness, reduce bias, and foster innovation, all of which contribute to higher-quality outcomes in team-based nursing management [60, 71, 72].

Decision-making competency in nurse managers is not developed in isolation; it is shaped by a constellation of organizational, contextual, and personal factors. Our findings confirm that elements such as information technology, group dynamics, and institutional culture directly modulate how this competency is expressed in real-world settings [71, 73]. In particular, the availability and usability of information systems significantly reduce the cognitive demands of decision-making by facilitating data processing and interpretation at scale [64, 68, 71].

Beyond structural support, strategic interventions are essential to strengthen this competency. Improving access to risk management systems, enhancing nurse managers' literacy in data use, and fostering a culture that values managerial creativity are key enablers of competent, responsive decision-making [74–76]. These strategies are most effective when aligned with organizational conditions that promote innovation and empower autonomy. By supporting the integration of technical tools with human judgment, they allow nurse managers to make informed, agile decisions that are attuned to the complexity of healthcare environments [77, 78].

Enabling and constraining factors shape the development and expression of decision-making competency in nurse managers. Among the key facilitators identified, access to electronic risk management systems, technological literacy, particularly in data handling, and a creative organizational culture stand out as crucial supports. These elements collectively enhance decision quality, reduce uncertainty, and empower adaptive leadership in complex settings.

In contrast, stress and the structural complexity of healthcare environments remain significant barriers. These findings reinforce the importance of designing work environments that reduce cognitive overload and support sustained attention, reflective reasoning, and informed action [79–83]. Cultivating a culture that encourages data-informed decision-making and critical thinking is essential to mitigate risk and build resilience in leadership roles.

Strengthening decision-making competency through targeted interventions and supportive environments can enhance organizational agility, promote professional development, and improve managerial effectiveness and care outcomes.

Enhancing decision-making competency in nurse managers has clear implications for improving the quality and safety of patient care. When this competency is well developed, nurse managers are better equipped to lead in complex environments, respond effectively to uncertainty, and guide clinical teams with clarity and accountability.

The results of this review offer a foundation for designing simulation-based training interventions that mirror the complexity of real-world decision-making. Simulation provides a safe space for nurse managers to engage with high-stakes' scenarios, apply critical thinking, and receive structured feedback. The 15 characteristics identified in this study can inform the construction of context-specific challenges that reflect the multifaceted demands of nursing leadership. This approach supports the assessment of decision-making competency and the design of individualized development strategies based on observed strengths and areas for improvement.

The findings of this review point to a promising opportunity to integrate emerging technologies, such as artificial intelligence and virtual reality, into the development, simulation, and assessment of decision-making competency in nurse managers. These tools can enhance training by replicating complex clinical scenarios, enabling adaptive feedback, and supporting individualized learning trajectories. They may also facilitate the operationalization of integrative models such as Park's, which optimize the interplay between quality, staffing, and cost-efficiency [84, 85].

At the same time, adopting such technologies must be guided by critical thinking and ethical deliberation. Approaches such as Park's sweet spot theory [86] provide a valuable framework for balancing innovation with professional judgment. However, the literature cautions against overreliance on automation, as it may undermine the reflective and context-sensitive elements of competent decision-making [85, 87]. In this regard, Park [88] also warns about the pedagogical and ethical risks of immersive technologies such as virtual reality, underscoring the importance of implementation strategies that prioritize educational quality, ethical responsibility, and learner well-being. This highlights the need for deliberate implementation strategies that align technological advancement with the foundational principles of nursing leadership.

### 4.1. Limitations

This review acknowledges that factors such as health policy, regulatory frameworks, institutional culture, and the nurse manager's role vary widely across healthcare systems. To address this, we included diverse studies to capture global perspectives and account for contextual variability. Nevertheless, the generalizability of findings may be constrained in settings where structural or cultural conditions differ significantly from those described in the reviewed literature.

This review included both studies explicitly focused on decision-making characteristics and broader research that, while not directly addressing these features, offered relevant insights through correlational and intervention designs. Although this methodological breadth enhances the comprehensiveness of our findings, it also introduces variability in the depth and specificity of the evidence. In addition, the limited volume of existing research on this topic constrains the generalizability of our conclusions. Nevertheless, the conceptual structure outlined in this study provides a strong foundation for future research aimed at refining, validating, and applying the decision-making competency framework in diverse healthcare contexts.

## 5. Conclusions

This study identifies and describes the key characteristics of decision-making competency for nurse managers, emphasizing the critical importance of values and ethics, information processing, systems and data management, and risk management. These aspects are fundamental to the development of this competency. Technology plays a pivotal role in mitigating the information overload faced by nurse managers, acting as a crucial facilitator by improving access to and analysis of information, thus enhancing the quality and efficiency of decisions.

The findings underscore the diversity of decision-making approaches that must be adapted to various contexts, highlighting the need to foster cultures conducive to creativity and innovation to navigate different environments effectively. In addition, the research demonstrates how both organizational and contextual factors significantly influence decision-making, underscoring the need for nurse managers to create work environments that minimize cognitive load and improve the construction of knowledge and understanding of data. Strategies identified to improve decision-making underscore the importance of improving access to and usability of risk management systems and require nurse managers to develop technological competencies to strengthen decision-making.

Electronic risk management systems are identified as the main facilitators of decision-making. This indicates that nurse managers must strengthen their capacity for electronic data management, supported by a culture of creativity. In contrast, stress and the complexity of the healthcare environment are highlighted as the main barriers, pointing to nurse managers' management of cognitive load as a crucial element in decision-making.

### 5.1. Implications for the Nurse Manager

This review provides a robust foundation for designing targeted training interventions to strengthen decision-making competency in nurse managers. Characterizing this competency supports the development of realistic and context-specific simulation scenarios, including those using virtual reality, to practice complex decision-making in controlled environments safely. Furthermore, the findings contribute to a better understanding of how decision-making competency intersects with leadership development, particularly in shared decision-making, change management, and innovation. This is especially relevant in dynamic healthcare settings where nurse managers must lead adaptive, evidence-based responses to organizational challenges.

## Figures and Tables

**Table 1:** Search strategy.

Database 2009–2025	Search terms
WOS	TS=(“head nurs^∗^” OR “frontline manager” OR “nurs^∗^ director” OR “nurs^∗^ manag^∗^” OR “first line nurs^∗^ manage^∗^” OR “nurse supervisor” OR “nursing program manager” OR “nurse unit manager” OR “chief nurse executive” OR “nurs^∗^ administrat^∗^” OR “director of nurs^∗^” OR “nurs^∗^ executiv^∗^”) AND TS = (“decision making” AND competenc^∗^)
SCOPUS	TITLE-ABS-KEY (“nurs^∗^ manag^∗^” OR “head nurs^∗^” OR “frontline manager” OR “nurs^∗^ director” OR “first line nurs^∗^ manage^∗^” OR “nurse supervisor” OR “nursing program manager” OR “nurse unit manager” OR “chief nurse executive” OR “nurs^∗^ administrat^∗^” OR “director of nurs^∗^” OR “nurs^∗^ executiv^∗^”) AND TITLE-ABS-KEY (“decision making” AND “competenc^∗^”)
PUBMED	(“Nurse administrators” [Majr] OR “nursing, supervisory” [Majr]) AND (“decision making”[Majr] OR “competenc^∗^”[All fields])
CINAHL	([TI “decision making” OR AB “decision making”] AND [TI competenc^∗^ OR AB competenc^∗^]) AND (TI [“nurs^∗^ manag^∗^” OR “nurse supervisor” OR “nurse unit manager” OR “chief nurse executive” OR “nurs^∗^ administrat^∗^” OR “director of nurs^∗^” OR “head nurs^∗^” OR “nurs^∗^ executiv^∗^”] OR AB [“nurs^∗^ manag^∗^” OR “nurse supervisor” OR “nurse unit manager” OR “chief nurse executive” OR “nurs^∗^ administrat^∗^” OR “director of nurs^∗^” OR “head nurs^∗^” OR “nurs^∗^ executiv^∗^”] OR MH “nursing administrator”)

*Note:* Fuente: own elaboration.

**Table 2:** Quality assessment tool adapted from Hölbl et al. [37].

Domain	Indicator (0–2)
Q1-Are the nurse manager's decision-making characteristics described?	No–moderately–yes
Q2-Are the research objectives clearly outlined?	No–moderately–yes
Q3-Are the main contributions well described in the nurse management's decision-making?	No–moderately–yes
Q4-How appropriate is the problem–solution fit?	No–moderately–yes
Q5-Are the proposed solutions feasible (scalable, economical, and implementable)?	No–moderately–yes

*Note: Source:* own elaboration based on bibliography.

**Table 3:** Clustering and frequency of characteristics related to nurse managers' decision-making competency.

Characteristics	Category description	Abs. Fr. (*n*)	Rel. Fr. (%)
Professional values and ethics	The ethical principles and values guide professional behavior and decision-making, reflecting a commitment to quality and integrity	22	13.10
Professional development and feedback	Continuous professional growth through self-evaluation and feedback to consistently improve decision-making practices	21	12.50
Communication and collaboration	The ability to effectively communicate and collaborate with colleagues and users of the health system to improve decision-making and quality of care	17	10.12
Information processing	The cognitive process of searching for, identifying, and utilizing relevant information to formulate and substantiate evidence-based informed decisions	16	9.52
Leadership and role-specific skills	The specific leadership and management skills are necessary to influence decision-making and direct teams within a healthcare setting	15	8.93
Risk management	The assessment and management of risks, adaptation and resilience to change in uncertain situations, and maintaining a balance between authority and compassion	15	8.93
Systems and data management	The systematic organization and management of clinical data and support resources for effective decision-making and efficient patient care administration	14	8.33
Organizational capacity and support	The ability of an organization to provide support and make decisive decisions that strengthen its operational capacity and human resources	12	7.14
Clinical application and confidence	The practical application of clinical knowledge with increasing confidence in decision-making, especially in emergency contexts	8	4.76
Clinical context and patient focus	The focus is on providing patient-centered care and maintaining clinical competence during critical healthcare situations	6	3.57
Realism and practical application	The ability to apply theoretical knowledge to practical scenarios realistically and effectively, enhancing preparation and critical thinking	6	3.57
Ethical use and assessment of information	The integrity and intellectual respect in handling information ensure decisions align with beneficence, autonomy, and justice principles	5	2.98
Time and complexity management	The skill to manage time and navigate the complexity of decisions in a dynamic and often unpredictable environment	5	2.98
Information management hierarchy	The hierarchical organization and management of information at various levels ensure the integrity and accessibility of information within the organization	3	1.79
Decision-making under pressure	The ability to maintain calm and clarity, effectively manage time, and determine critical moments for decision-making in high-pressure situations	3	1.79

*Note: Source:* own elaboration based on bibliography.

**Table 4:** Classification of decision-making characteristics into dimensions.

Dimension	Definition	Characteristics
Strategic and ethical decision-making	Integrating ethical principles, critical thinking, and situational adaptability ensures sound and responsible decision-making in healthcare management. This dimension reflects a nurse manager's ability to assess complex situations, uphold ethical standards, and manage uncertainty	Professional values and ethics Decision-making under pressure Risk management Time and complexity management

Information synthesis and application	The ability to systematically gather, process, and apply information to support evidence-based decision-making, ensuring data-driven and informed managerial actions	Information processing Systems and data management Realism and practical application Information management hierarchy

Organizational and interpersonal decision-making	The ability to effectively communicate, collaborate, and utilize organizational resources to optimize decision-making processes in dynamic healthcare settings	Communication and collaboration Organizational capacity and support Clinical context and patient focus

Professional growth and reflective decision-making	Continued refinement of decision-making competency through self-assessment, feedback, and professional development leads to increased confidence and expertise in managerial decisions	Professional development and feedback Clinical application and confidence Ethical use and assessment of information

*Note: Source:* Own elaboration based on bibliography.

## Data Availability

The datasets generated and/or analyzed during the current study are available from the corresponding author upon request.

## Supporting Information

Supporting File 1: Table S1: Quality assessment. Table S2: Description of the reviewed articles and their relevance to nurse managers' decision-making competency. Table S3: Decision-making styles. Table S4: Factors influencing nurse managers' decision-making competency and their consequences. Table S5: Impact of technology on decision-making. Table S6: Integrated summary of barriers, facilitators, and improvement strategies for nurse managers' decision-making competency.
