# Supplementary material for: Characterizing the Decision-Making Competency of Nurse Managers: A Scoping Review
Source: J Nurs Manag. 2025 Jun 10;2025:2771210. doi: 10.1155/jonm/2771210 (PMC12173547; doi:10.1155/jonm/2771210)
Supplement: Supporting Information — Additional supporting information can be found online in the Supporting Information section. [file 2771210.f1.docx]

**Supplementary File 1**

Table S1. Quality Assessment

| ID | Q1 | Q2 | Q3 | Q4 | Q5 | Total Score | Mean  Total score (SD) |
| --- | --- | --- | --- | --- | --- | --- | --- |
| 1 | 2 | 2 | 2 | 2 | 1 | 9 |  |
| 2 | 2 | 2 | 0 | 2 | 0 | 6 |  |
| 3 | 2 | 2 | 1 | 0 | 2 | 7 |  |
| 4 | 2 | 2 | 2 | 0 | 0 | 6 |  |
| 5 | 2 | 2 | 2 | 2 | 2 | 10 |  |
| 6 | 2 | 2 | 2 | 2 | 2 | 10 |  |
| 7 | 2 | 2 | 0 | 1 | 2 | 7 |  |
| 8 | 2 | 0 | 2 | 2 | 2 | 8 |  |
| 9 | 2 | 2 | 2 | 1 | 2 | 9 |  |
| 10 | 2 | 2 | 2 | 2 | 1 | 9 |  |
| 11 | 2 | 2 | 2 | 1 | 1 | 8 |  |
| 12 | 2 | 2 | 1 | 1 | 1 | 7 |  |
| 13 | 2 | 2 | 0 | 1 | 0 | 5 |  |
| 14 | 2 | 2 | 1 | 1 | 1 | 7 |  |
| 15 | 2 | 2 | 1 | 1 | 1 | 7 |  |
| 16 | 2 | 2 | 2 | 2 | 2 | 10 |  |
| 17 | 2 | 2 | 2 | 2 | 2 | 10 |  |
| 18 | 2 | 2 | 2 | 2 | 2 | 10 |  |
| 19 | 2 | 2 | 2 | 2 | 2 | 10 |  |
| 20 | 2 | 2 | 2 | 2 | 2 | 10 |  |
| 21 | 2 | 2 | 0 | 1 | 0 | 5 |  |
| 22 | 2 | 2 | 1 | 1 | 1 | 7 |  |
| 23 | 2 | 2 | 2 | 2 | 2 | 10 |  |
| 24 | 2 | 2 | 2 | 1 | 2 | 9 |  |
| 25 | 2 | 2 | 2 | 1 | 2 | 9 |  |
| Mean (SD) | 2 | 1.92 | 1.48 | 1.4 | 1.4 | 8.2 | 8.2 |
| SD | 0.00 | 0.39 | 0.75 | 0.63 | 0.75 | 1.65 | 1.65 |

Table S2. Description of the reviewed articles and their relevance to nurse managers' decision-making competency

| Author/Year | Method | Description/context | Key findings | Limitations |
| --- | --- | --- | --- | --- |
| Toren et al., 2010 [52] | Case analysis with ethical tool | Application of an ethical decision-making model to a nurse manager's real dilemma involving confidentiality and staff safety (Israel) | Demonstrated how structured ethical reasoning supports balanced decisions in complex situations. Highlighted moral responsibility, stakeholder consultation, and alignment with professional values. | Single-case design; limited generalizability. |
| Omoike et al., 2011 [53] | Mixed-methods study | Assessment of self-perceived leadership competencies among 41 nurse leaders using a survey and focus group discussions. (USA) | Nurse leaders perceived themselves as competent in technical areas (e.g., budgeting, staffing, communication), yet less competent in strategic thinking and relationship building—identified gaps between perceived and expected competency levels in decision-making. | Small, non-randomized sample; limited generalizability due to single-institution context. |
| Furukawa & Cunha, 2011 [54] | Qualitative descriptive study | An exploratory study using focus groups with nurse managers from a hospital in São Paulo to identify key managerial competencies for professional practice. | Identified strategic vision, decision-making, teamwork, communication, and leadership as essential competencies for nurse managers' effectiveness. | The scope was limited to a single institution, which may affect generalizability; there is an absence of quantitative validation of the identified competencies. |
| Piper & Czekanski, 2012 [38] | Role–playing within a nursing management program for graduates | Role–playing exercise to simulate the type of ethical and informed decision–making required in nurse managers, in a nursing management course. (USA) | Identified listening ability, idea reaffirmation, and synthesis as key components of nurse managers' decision-making competency, particularly in ethical decision-making contexts | Not described |
| Shirey et al., 2013 [39] | Qualitative descriptive study with interviews | Interviews with 21 nurse managers from three hospitals on decision–making in contexts of stress and complexity. (USA) | Identified a cognitive model that guides nurse managers' decision-making competency under stressful conditions. This model highlights adaptive thinking, resilience, and prioritization as critical characteristics in complex healthcare environments. | Intentional sampling techniques limited the transferability of the study's results to other populations. |
| Eduardo et al., 2015 [40] | Action research, with questionnaires and semi-structured interviews | Analyzing the decision-making model of managerial nurses through hospital nursing managers. (Brazil) | The study emphasizes the need to integrate structured decision-making models into nurse managers' competency framework to enhance the' effectiveness, consistency, and impact of their managerial decisions. It highlights the role of theoretical frameworks in strengthening decision-making competency. | Not described |
| Zydziunaite et al., 2015 [41] | Qualitative study, with data content analysis. | Forty-nine nurses in hospitals and health centers performed written reflections on decision–making and ethical dilemmas. (Lithuania) | The study identifies decision-making competency gaps among nurse managers when addressing ethical dilemmas, highlighting discrepancies between expected ethical standards and practical decision-making constraints. It underscores the need for enhanced training and support systems to strengthen ethical decision-making skills. | Data collection technique. |
| Luo et al., 2016 [55] | Qualitative exploratory study | In-depth interviews were conducted with 15 nurse managers from five tertiary hospitals in China to explore perceived competencies required for effective nursing management. | The study identified multiple competencies critical to decision-making in nurse managers, including analytical thinking, information processing, emotional self-regulation, ethical reasoning, organizational awareness, and leadership skills. | Conducted in a single region of China; limited transferability due to cultural and systemic specificity. |
| Jensen et al., 2016 [42] | Survey study | Based on a model (TIGER), a questionnaire was developed for 32 nurses specialized in health informatics and nursing management (Brazil) | The study highlights the role of informatics competencies in enhancing decision-making among nurse managers, particularly in evidence-based practice, data-driven decision-making, and practical information management. Findings suggest that technological proficiency is a key facilitator in nurse managers' decision-making processes. | Not described |
| Siirala et al., 2016 [43] | Qualitative descriptive design | Data were collected from 20 nurse managers using the think-aloud method during the busiest work hours and analyzed through thematic content analysis. (Finland) | The study highlights that decision-making among nurse managers is highly susceptible to workplace interruptions, impacting their ability to analyze information effectively, prioritize tasks, and make timely decisions. Findings suggest that minimizing external disruptions may be crucial in enhancing decision-making competency in high-pressure healthcare environments | Not described |
| Islam et al., 2018 [44] | Qualitative research | Information requirements of nursing managers, risk management practices, and influences on decision making when interacting with an electronic risk management system. (Australia) | Nurse managers' decision-making processes are shaped by their ability to navigate electronic risk management systems, assess data reliability, and effectively communicate risk-related information. The study identifies three key themes influencing decision-making competency: system navigation, data trust, and communication and feedback. Findings highlight the importance of technological proficiency as a facilitator of decision-making competency in nursing management. | Not described |
| Moghaddam et al., 2019 [56] | Mixed-methods: Delphi technique, analytic hierarchy process, tool development, and validation | Development and validation of a model and tool to assess the managerial competencies of head nurses in Iranian hospitals, including planning, organizing, leadership, and control tasks. | Identified 27 key managerial competencies grouped into four main functional domains. Among them, strategic thinking, critical thinking, resource allocation, and performance evaluation were emphasized. A validated assessment tool was created with high reliability and content validity. | Cross-sectional design; the model was developed and tested within one national healthcare context, limiting its immediate generalizability to other settings. |
| Roshanzadeh et al., 2020 [29] | Qualitative analysis with in-depth interviews | Interviews with 19 nurse managers from different hospitals were conducted through semi–structured interviews and subsequent content transcription. (Iran) | Nurse managers recognize ethical decision-making as a fundamental component of their professional role; however, their competency in this area varies. The study highlights the need for further development in ethical reasoning, moral judgment, and applying ethical frameworks when making managerial decisions. Findings suggest that personal values, institutional policies, and organizational culture influence decision-making competency in ethical contexts. | Not described |
| Gunawan et al., 2020 [57] | Cross-sectional study | A survey of 233 first-line nurse managers (FLNMs) from 13 public hospitals in Indonesia was conducted to assess self-perceived managerial competence using a validated scale (I-FLNMMCS). | FLNMs in larger, Ministry of Health–owned hospitals demonstrated significantly higher self-perceived competence, particularly in leadership, self-management, informatics, and financial domains. Managerial training and length of service were influential. | Convenience sampling and self-assessment may introduce bias, and generalizability is limited beyond Indonesia. |
| Chisengantambu-Winters et al., 2020 [8] | Structured interviews, hermeneutic principles | Exploring decision–making among nurse managers in rural and regional health settings in South Australia | Nurse managers in rural and regional healthcare settings face unique decision-making challenges due to resource limitations, workforce shortages, and dependency on external support systems. The study proposes the Decision-Making Dependency (DMD) model, which outlines how organizational and environmental constraints shape managerial decision-making. Findings suggest that decision-making competency in these settings requires adaptability, resilience, and enhanced problem-solving skills to navigate structural limitations effectively | Not described |
| Ocho et al., 2021 [58] | Mixed-methods study | A study involving 126 nurses promoted to leadership positions across four Caribbean countries. Data collection included questionnaires and focus groups on competencies and challenges in transitioning to leadership roles. (Caribbean) | Key competencies identified included leadership, motivation, teamwork, delegation, emotional intelligence, and networking. Reported challenges included nepotism, lack of formal preparation, and conflicts in managerial practice. | The study was approved in only four countries. A limited number of focus groups restricts the generalizability of the qualitative findings. |
| Roshanzadeh et al., 2021[45] | Qualitative content analysis | Explores nursing managers' experiences with courage in ethical decision–making in Iran, using in–depth interviews with 19 managers | Nurse managers demonstrate courage in ethical decision-making through decisiveness and a strong sense of professional obligation. The findings highlight assertiveness and commitment to ethical principles as essential to decision-making competency, particularly when addressing ethical dilemmas in healthcare settings. This study underscores the importance of moral reasoning, confidence, and professional integrity in shaping decision-making competency among nurse managers. | Focused on experiences in specific regions |
| Manlangit et al., 2022 [31] | Quantitative correlational study | Surveyed 80 nurse leaders in a university hospital in the Philippines to examine the relationship between cultural competence and decision-making skills. | The study found a significant positive relationship between cultural competence and decision-making among nurse leaders. Key competencies include cultural awareness, sensitivity, behavior, critical thinking, and ethical awareness in decision-making processes. | Small sample size and limited generalizability due to single-site data collection. |
| AlAmer, 2023 [1] | Multi–center cross–sectional study | Investigates the relationship between decision–making styles and managerial creativity among nursing managers in Saudi Arabia. | The findings reveal that distinct decision-making styles influence decision-making competency. A rational decision-making style enhances creativity, while avoidant and dependent styles hinder it. This study highlights the importance of analytical thinking and autonomy as essential characteristics of decision-making competency in nurse managers. The results suggest that fostering rational decision-making may improve managerial effectiveness. | Not described |
| Tazebew et al., 2023[46] | Cross–sectional study | Examining nurse managers' involvement in decision–making within government hospitals in Addis Ababa, Ethiopia. | The study highlights variability in decision-making competency among nurse managers. Nurse managers in matron positions exhibit higher decision-making involvement than head nurses, suggesting that experience and hierarchical position influence decision-making competency development. Additionally, managerial support and structured feedback mechanisms facilitate nurse managers' participation in decision-making processes. | Not described |
| Burrell et al., 2023[47] | Impact of simulation on nursing students' management of oncological emergencies. | Impact of simulation on the management of oncologic emergencies by nursing students. (USA) | The results indicate that simulation-based learning enhances decision-making competency by improving confidence, reducing anxiety, and increasing overall learning satisfaction. This suggests experiential learning strategies can strengthen decision-making abilities in high-pressure healthcare scenarios, a crucial component of nurse managers' training and professional development. | Small sample size |
| Kusakli & Sönmez, 2024 [48] | Randomized controlled trial | Intervention in a tertiary hospital in Istanbul (Türkiye) used case-based training to enhance nurse managers' problem-solving and decision-making skills; evaluation included self-assessments and ratings from subordinates. | The training significantly improved nurse managers' decision-making and problem-solving abilities across multiple styles, with measurable effects confirmed by self and subordinate evaluations after three months. | Conducted in a single hospital; voluntary participation may introduce bias despite randomization. |
| Abeje et al., 2025 [49] | Cross-sectional quantitative study | A survey was conducted in 14 government hospitals in South Wollo Zone (Ethiopia) with 168 nurse managers to assess the prevalence of poor decision-making and identify influencing factors. | 35.7% of nurse managers exhibited poor decision-making. Self-confidence, receiving feedback from colleagues, and managerial support were significantly associated with improved decision-making competency. | Cross-sectional design limits causal inference; it lacks qualitative insight into decision-making experiences. |
| Millard et al., 2024 [50] | Prospective pre-post intervention study | Implementation and evaluation of a Charge Nurse Readiness Program in a cardiothoracic ICU at an academic hospital in the U.S. to improve nurse manager decision-making and collaboration. | Using a standardized tool and structured process improved nurse managers' decision-making, transparency in charge of nurse selection, and staff satisfaction. Peer and self-assessments enhanced feedback and readiness. | Single-unit study in one institution; generalizability may be limited. |
| Hossny & Alotaibi, 2024 [51] | Cross-sectional quantitative study | A study was conducted in four university hospitals with 229 nurse managers to examine the relationship between dominant decision-making styles and managerial creativity. | Rational style was most dominant and positively associated with creativity. Dependent style was the second most used and negatively associated. Intuitive style had no significant association. Creativity levels were generally high. | Cross-sectional design limits causality; the majority female sample limits generalizability due to gender imbalance in leadership roles. |

Source: own elaboration based on bibliography

Table S3. Decision-making styles

| Author/Year | Decision-Making Style |
| --- | --- |
| Siirala et al., 2016 [43] | Tactical and Operational |
| (Chisengantambu-Winters et al., 2020 [8] | Dependent |
| Roshanzadeh et al., 2021 [45] | Ethical based on courage |
| AlAmer, 2023 [1] | Rational, Avoidant, Dependent, Intuitive, Spontaneous |
| Tazebew et al., 2023 [46] | Participative |
| Hossny & Alotaibi, 2024 [51] | Rational, Dependent, Intuitive |

Source: own elaboration based on bibliography.

Table S4. Factors Influencing Nurse Managers' Decision-Making Competency and Their Consequences

| Author/Year | Contextual and Organizational Factors | Personal Factors | Consequences for Patients | Consequences for Staff | Consequences for the Organization |
| --- | --- | --- | --- | --- | --- |
| Toren et al., 2010 [52] | Organizational policies, legal and ethical regulations, and a lack of reporting tools | Professional judgment, emotional burden, personal ethical values, loyalty dilemmas | Improved patient outcomes through effective staffing decisions | Increased autonomy and decision-making capacity among nurse managers | Enhanced organizational efficiency through clarified managerial roles |
| Omoike et al., 2011 [53] | Leadership development program structure; institutional investment in training | Self-assessment of leadership competencies; perceived skill level gaps | – | Enhanced leadership self-awareness; improved perception of preparedness | Demonstrated institutional value in leadership development initiatives |
| Furukawa et al., 2011 [54] | Organizational expectations, evaluation by superiors, selection criteria, and institutional role modeling | Leadership skills; communication ability; decision-making capacity; teamwork; personal initiative; ethical commitment | – | – | – |
| Piper & Czekanski, 2012 [38] | Use of information technologies, team dynamics, and course structure | Self–perceived confidence and competence; anxiety in clinical decisions | Improvement in oncological emergency management | Increase in confidence and competence | Better preparation of nursing students in practice |
| Shirey et al., 2013 [39] | Work stress and complexity, organizational support | Role experience and personal characteristics of the decision-maker | – | Stress related to decision–making | Influence of organizational context on decision–making |
| Jensen et al., 2016 [42] | Implementation of informational literacy competencies in nursing education | Competencies in informatics and information for decision-making | – | Improvement in decision–making based on informatics competencies | Incorporation of informatics competencies in nursing education |
| Luo et al., 2016 [55] | Lack of clear role boundaries, expectations from upper management, and hierarchical authority | Professional values, communication style, self-confidence, and cultural background | – | Greater clarity in professional identity; enhanced communication and leadership capacity | Improved organizational awareness of the need to support nurse manager roles |
| Siirala et al., 2016 [43] | Daily operational management in perioperative settings: need for ad hoc information | – | Adherence to ethical principles | Improvement in ethical sensitivity and assertiveness | Encouragement of ethical training in management |
| Islam et al., 2018 [44] | Electronic risk management systems: organizational barriers | Information management skills; individual barriers | Optimization of risk management systems use | Identification of individual and organizational barriers | Need for adjustments in electronic management systems |
| Moghaddam et al., 2019 [56] | Hospital organizational structure, availability of resources, and need for performance evaluation models | Strategic thinking; professionalism; critical thinking; creativity; emotional self-management | – | – | – |
| Gunawan et al., 2020 [57] | Cultural diversity in staff composition, hierarchical organizational structure, and absence of role-specific ethical guidelines | Ethical sensitivity, individual values, and personal moral beliefs | – | – | – |
| Roshanzadeh et al., 2020 [29] | – | Ethical sensitivity; assertiveness; commitment | Adherence to ethical principles | Improvement in ethical sensitivity and assertiveness | Encouragement of ethical training in management |
| Chisengantambu-Winters et al., 2020 [8] | Multiplicity of roles, environment, and resources | Consultation: personal characteristics of the decision–maker | – | Empowerment in decision–making through the DMD model | Identification of factors affecting decision–making in rural health |
| Ocho et al., 2021 [58] | Nepotism, lack of formal preparation, and role ambiguity | Emotional intelligence, delegation capacity, and motivation | – | Improved self-awareness and confidence among novice nurse leaders | Identified the need for structured preparation programs and organizational accountability |
| Roshanzadeh et al., 2021 [45] | – | Ethical courage, education, and awareness of ethical issues | – | Strengthening ethical courage | Promotion of awareness on ethical issues |
| Manlangit et al., 2022 [31] | Cultural incongruence in healthcare settings: lack of organizational support for culturally informed decisions | Personal cultural competence, ethical sensitivity, and confidence in intercultural decision-making | Improved patient satisfaction through culturally competent care delivery | Increased confidence in managing cross-cultural situations | Recognition of the importance of cultural competence in organizational decision-making |
| AlAmer, 2023 [1] | Organizational culture, managerial support | Decision–making styles: managerial creativity | – | Relationship between decision–making styles and managerial creativity | Influence of organizational culture on creativity |
| Tazebew et al., 2023 [46] | Decision–making participation; managerial support | Matron position: receiving feedback | – | Greater involvement in decision–making | Improvement in managerial support and feedback |
| Burrell et al., 2023 [47] | Family inclusion in emergencies: realism of simulations | Self–confidence in decision–making; decreased anxiety in emergencies | Increase in learning satisfaction regarding emergency management | Decrease in anxiety in emergencies | Improvement in educational quality for emergency management |
| Millard et al., 2024 [50] | Standardized decision-support tools, structured role preparation, and institutional transparency | Peer and self-assessment, individual readiness, and self-perception | – | Increased readiness, confidence, and satisfaction among charge nurses | Greater transparency in charge nurse selection, improved decision-making processes |
| Hossny & Alotaibi, 2024 [51] | – | Dominant decision-making style; managerial creativity | – | Increased awareness of how decision-making style impacts creativity and performance | Identification of decision-making profiles is helpful for leadership development |
| Abeje et al., 2025 [49] | Managerial support; hospital-level variability | Self-confidence, receiving feedback from colleagues | – | Increased awareness of personal and organizational factors influencing decision-making; recognition of the need for self-improvement | – |

Source: own elaboration based on bibliography.

Table S5. Impact of Technology on Decision Making

| Authors/Year | Evaluated Technologies | Support in Decision-Making | Challenges in Decision-Making |
| --- | --- | --- | --- |
| Jensen et al., 2016 [42] | Information literacy competencies | Improve evidence–based decision–making skills | Requires effective integration into education |
| Islam et al., 2018 [44] | Electronic risk management systems | Facilitate the management and analysis of incidents | Organizational and usability barriers |
| Chisengantambu-Winters et al., 2020 [8] | Decision–making dependency models (DMD) | Support the structuring of complex decisions | Need for adaptation to local contexts |

Source: own elaboration based on bibliography.

Table S6. Integrated Summary of Barriers, Facilitators, and Improvement Strategies for Nurse Managers' Decision-Making Competency

| Author/Year | Facilitators for Decision Making | Barriers to Decision Making | Improvement Strategies |
| --- | --- | --- | --- |
| Toren et al., 2010 [52] | Ethical sensitivity, professional values, sense of responsibility, and autonomy in decision making | Lack of support from hospital management; tension between organizational goals and ethical standards | Promote autonomy, reinforce professional ethics, and ensure managerial training on ethical sensitivity and responsibility. |
| Omoike et al., 2011 [53] | Continuing education, leadership development programs, and a supportive peer environment | Lack of formal leadership preparation; inconsistent access to training opportunities | Provide leadership development programs and continuous professional education to enhance decision-making capacity among nurse leaders. |
| Furukawa et al., 2011 [54] | Alignment of personal and institutional values; participation in decision-making; organizational support | Lack of recognition of nurses' leadership role; limited involvement in institutional planning processes | Develop leadership, focus on patient care, teamwork, and decision-making competencies through targeted training; align managerial profiles with organizational expectations. |
| Shirey et al., 2013 [39] | Organizational support, role experience | Stress and job complexity, lack of organizational support | Provide organizational support and opportunities for continuous professional development to manage stress and job complexity. |
| Luo et al., 2016 [55] | Supportive hospital culture; alignment between training and real management demands | Lack of managerial autonomy; insufficient experience in conflict resolution and team leadership | Develop competency-based training programs tailored to real management challenges; emphasize strategic thinking and emotional intelligence for leadership growth. |
| Jensen et al., 2016 [42] | Integration of informatics competencies in education | Lack of adequate training in technology competencies | Integrate information literacy competencies into nursing education to strengthen evidence–based decision making. |
| Islam et al., 2018 [44] | Access to electronic risk management systems | Organizational and usability barriers in electronic systems | Improve access and usability of electronic risk management systems. |
| Moghaddam et al., 2019 [56] | – | – | Develop and apply a validated assessment model of managerial competencies to identify training needs and guide continuous performance improvement among head nurses. |
| Roshanzadeh et al., 2020 [29] | Awareness and training in ethical principles | Lack of assertiveness and ethical commitment among staff | Promote education and empowerment on ethical principles to enhance assertiveness and commitment in ethical decisions. |
| Chisengantambu-Winters et al., 2020 [8] | Use of decision-making models to structure complex decisions | Need to adapt models to specific local contexts | Implement decision-making models like the DMD to structure and adapt complex decisions to local contexts. |
| Gunawan et al., 2020 [57] | Ethical sensitivity, support from nursing education, and awareness of cultural values | Conflicting cultural values, limited institutional support for ethical decision-making, and a lack of training in moral reasoning | Strengthen nurse managers' decision-making competency through moral sensitivity training, promotion of ethical awareness, and leadership development programs tailored to culturally diverse settings. |
| Ocho et al., 2021 [58] | Leadership motivation, emotional intelligence, peer support, and collaborative environments | Nepotism, lack of formal preparation, absence of structured transition programs, and internal conflicts in team management | Develop formal leadership training programs tailored to newly appointed nurse managers, focusing on essential decision-making competencies such as delegation, motivation, and teamwork. |
| Manlangit et al., 2022 [31] | Development of cultural self-awareness, respect for diversity, collaborative leadership, value-aligned practice | Lack of cultural training, value conflict, ethnocentric attitudes, difficulty integrating diverse perspectives | Integrate cultural competence development programs focused on awareness, knowledge, and skill-building to enhance nurse leaders' decision-making in diverse healthcare settings |
| AlAmer, 2023 [1] | An organizational culture that values creativity | Preferences for decision-making styles that limit creativity | Promote an organizational culture that values managerial creativity and supports diverse decision–making styles. |
| Tazebew et al., 2023 [46] | Managerial support, positive feedback | Lack of staff involvement in decision-making processes | Increase staff involvement in decision-making through positive feedback and managerial support. |
| Kusakli & Sönmez, 2024 [48] | Structured training based on case scenarios; evaluation from multiple perspectives | Limited prior training; variability in baseline decision-making skills | Implement case-based training programs to enhance nurse managers' problem-solving and decision-making competencies. |
| Millard et al., 2024[50] | Structured training, peer and self-assessment, standardized tools, and mentorship | – | Implement a structured Charge Nurse Readiness Program with standardized tools, peer/self-assessment, and mentorship to improve decision-making capacity and leadership preparedness. |
| Hossny & Alotaibi, 2024 [51] | Rational decision-making style; managerial creativity | Dependent decision-making style; lack of strategic decisional autonomy | Promote rational decision-making styles and managerial creativity as key elements for leadership development. |
| Abeje et al., 2025 [49] | Feedback from colleagues, managerial support, and self-confidence | Limited managerial support, low self-confidence, and an insufficient feedback culture | Strengthen nurse managers' self-confidence and promote structured feedback and managerial support to enhance decision-making competency. |
